# Cryo-EM Structure of a Type I Anti-Prothrombin Antibody Reveals a Novel Kringle-1 Epitope and Its Functional Impact

**DOI:** 10.1063/4.0000970

**Published:** 2025-10-27

**Authors:** Suresh Kumar, Nicola Pozzi

**Affiliations:** Saint Louis University, Saint Louis, Missouri, USA

## Abstract

**Introduction.:**

Antiphospholipid syndrome (APS) is an autoimmune disorder characterized by the presence of antiphospholipid antibodies (aPLs) that disrupt coagulation and increase thrombosis risk. Prothrombin is a key antigen for aPL, and we identified two subpopulations of anti- prothrombin antibodies, Type-I and Type-II in APS. Type-I antibodies selectively recognize the open conformation of prothrombin, while Type-II binds both open and closed forms. Previously, we identified the epitope for the Type-I monoclonal antibody POmAb within kringle-1 (K1), specifically at residues R90-Y93. However, the binding site and functional effects of another Type-I antibody 5B10 remained unknown.

**Methods.:**

To define the structural and functional properties of 5B10, we determined its complex with prothrombin using cryogenic-electron microscopy (cryo-EM). The impact of 5B10 on prothrombin conformation and dynamics was assessed using single-molecule Förster resonance energy transfer (smFRET). Functional effects were evaluated through plasma coagulation assays, including prothrombin time (PT), activated partial thromboplastin time (aPTT), and dilute Russell viper venom time (dRVVT), as well as thrombin generation assay.

**Results.:**

The cryo-EM structure of 5B10 bound to prothrombin was resolved at 3.2 Å resolution, providing high-confidence assignment of intermolecular interactions. Unlike POmAb, which targets a linear epitope involving residues R90-Y93, 5B10 binds a conformational and discontinuous epitope in K1, primarily involving the 80- and 130-loops. Notably, weak density was observed for the Gla domain, while kringle-2 and the serine protease domain were undetectable, suggesting structural flexibility. smFRET analysis demonstrated that 5B10 shifts prothrombin toward the open conformation, while maintaining high dynamic mobility. Despite these differences in epitope recognition and binding orientation, 5B10 and POmAb exhibited nearly identical functional profiles in coagulation assays, acting as non-competitive inhibitors of thrombin generation. Conclusion. Our study reveals a novel Type I antibody epitope within K1 of prothrombin and highlights its structural and functional consequences. Despite binding distinct sites, 5B10 and POmAb share a common mechanism that interferes with thrombin generation, suggesting a broader paradigm for Type I antibody- mediated dysregulation in APS. These findings provide new insights into prothrombin-targeting autoantibodies and their role in APS pathophysiology.

